# Case Report: Recurrent gestation-limited intractable abdominal colic with elevated phthalate and paraben biomarkers: a hypothesis-generating observation

**DOI:** 10.3389/fmed.2026.1875138

**Published:** 2026-07-13

**Authors:** Suling Sun, Yadan Zou, Xiangyuan Liu, Sheng-Guang Li

**Affiliations:** 1 Department of Reproductive Medicine, Qingdao Lianchi Women & Infants Hospital Co., Ltd., Qingdao, China; 2 Department of Rheumatology and Immunology, Peking University International Hospital, Beijing, China

**Keywords:** case report, endocrine-disrupting chemicals, monoethyl phthalate, parabens, phthalates, pregnancy-specific abdominal colic

## Abstract

**Background:**

Recurrent, gestation-limited severe abdominal colic without a conventional diagnosis is rarely described. Endocrine-disrupting chemicals (EDCs) have been associated with adverse reproductive outcomes, but their relevance to recurrent pregnancy-specific gastrointestinal syndromes remains uncertain.

**Case presentation:**

A 35-year-old Chinese woman had five prior pregnancies; four were terminated because of recurrent severe lower abdominal colic, constipation, inability to maintain oral intake, and marked weight loss. Symptoms reproducibly began at 11–12 gestational weeks and resolved within 1–2 weeks after pregnancy termination. Extensive obstetric, gastrointestinal, vascular, autoimmune, genetic, and imaging evaluations were unrevealing. On 14 September 2024, first-morning urine biomonitoring by ultra-performance liquid chromatography–tandem mass spectrometry (UPLC–MS/MS) showed creatinine-adjusted monoethyl phthalate 2136.48 μg/g Cr, methylparaben 233.40 μg/g Cr, and propylparaben 53.80 μg/g Cr, all above the laboratory upper reference limits. A multidomain preconception program addressed avoidable EDC exposure, metabolic factors, immune-vascular factors, nutritional deficits, and gut dysbiosis. Repeat first-morning urine testing on 20 July 2025, during early pregnancy and before the historical 11–12-week symptom window, showed the three previously elevated biomarkers below the laboratory limits. The pregnancy was complicated by hyperemesis gravidarum and transient gestational thyrotoxicosis, but the prior intractable abdominal colic did not recur, and a healthy 3.6-kg female infant was delivered vaginally at term on 14 March 2026.

**Conclusion:**

Because biomarkers were not measured during the prior symptomatic pregnancies and the intervention was multifactorial, this case cannot establish temporality or causality. It supports targeted exposure history and selective EDC biomonitoring as a clinically modifiable, hypothesis-generating clue in carefully selected patients with recurrent, unexplained, pregnancy-specific morbidity after standard evaluation has been exhausted.

## Introduction

Severe abdominal pain during pregnancy usually prompts evaluation for obstetric emergencies, gastrointestinal disease, urinary tract disorders, vascular events, and inflammatory or autoimmune conditions. When symptoms recur at a reproducible gestational window, remain absent outside pregnancy, and resolve after pregnancy termination, the diagnostic problem may extend beyond a fixed structural lesion to pregnancy-specific maternal, placental, endocrine, vascular, or neurogastrointestinal mechanisms. Professional guidance also encourages clinicians to consider environmental toxicants during preconception and prenatal care because such exposures may contribute to adverse reproductive and developmental outcomes (1).

Environmental endocrine-disrupting chemicals (EDCs), including phthalates and parabens, are relevant but nonspecific exposures in this context. They may interfere with hormone signaling, metabolism, immune regulation, oxidative balance, and placental biology (2, 3). These compounds are commonly encountered through plastics, food-contact materials, fragrances, cosmetics, medications, and personal care products, and several urinary phthalate and paraben biomarkers have been associated with reproductive outcomes in epidemiologic studies, although findings remain heterogeneous and cannot be extrapolated directly to unusual pregnancy-limited gastrointestinal phenotypes (4–10).

We report a decade-long case of recurrent gestation-limited intractable abdominal colic leading to repeated pregnancy terminations, followed by detection of markedly elevated creatinine-adjusted monoethyl phthalate, methylparaben, and propylparaben, multidomain preconception intervention, biomarker decline, and a later term live birth without recurrence of the prior colic phenotype. This case is presented as a hypothesis-generating clinical observation rather than evidence of causality.

## Case presentation

### Patient information and prior obstetric phenotype

A 35-year-old Chinese woman, born in 1989 and working as a diagnostic sonographer, presented on 27 July 2024 for evaluation of recurrent pregnancy-specific intractable abdominal pain. She had been generally healthy, with no established chronic gastrointestinal, endocrine, autoimmune, or allergic disease. Menstrual cycles were regular. Before the index pregnancy, her obstetric history was recorded as gravida 5, para 0. Four pregnancies were terminated because of severe lower or left lower abdominal colic, constipation, inability to maintain oral intake, and marked weight loss. Symptoms typically appeared at 11–12 gestational weeks, were absent outside pregnancy, and resolved within 1–2 weeks after pregnancy termination. One pregnancy in 2016 was electively terminated at 6 weeks because of concern regarding paternal anti-hepatitis B medication exposure rather than maternal abdominal pain (Table 1).

**TABLE 1:** Obstetric history and pregnancy-specific symptom phenotype.

Gravida (year)	Onset of symptoms	Key clinical features	Diagnostic evaluations and interventions	Pregnancy outcome
G1 (2014)	11w+2d	Severe hyperemesis initially, followed by sudden unremitting post-prandial abdominal colic; severe weight loss from 54 to 39 kg.	Whole-abdominal contrast-enhanced CT at 19 weeks did not identify mesenteric arterial thrombosis or another explanatory lesion; post-abortion EGD/colonoscopy were not explanatory.	Induced abortion at 19w+3d due to maternal intolerance; pain resolved in approximately 2 weeks.
G2 (2016)	N/A	Asymptomatic.	N/A	Elective termination at 6w because of concern about paternal antiviral medication exposure.
G3 (2017)	12w	Recurrent severe lower abdominal colic; poor response to enema, anisodamine, and opioids.	Abdominal CT not explanatory; upper gastrointestinal radiography excluded hiatal hernia.	Induced abortion at 14w due to maternal intolerance and refractory pain.
G4 (2019)	11w+3d	Recurrent excruciating lower abdominal colic.	Extensive inter-conception workup at tertiary hospitals, including imaging, endoscopy, and autoimmune assessment, did not identify a cause.	Pregnancy termination due to refractory pain.
G5 (2021)	12w+3d	Recurrent intolerable lower abdominal colic.	No explanatory diagnosis documented.	Pregnancy termination due to refractory pain.
G6 (2025–2026)	No recurrent colic	Severe hyperemesis gravidarum with ketonuria, electrolyte disturbance, and transient gestational thyrotoxicosis; the historical colic phenotype did not recur.	Two hospitalizations; intravenous fluids, thiamine, antiemetics, electrolyte correction, and central venous access.	Term vaginal delivery of a healthy 3.6-kg female infant.

CT, computed tomography; EGD, esophagogastroduodenoscopy; HG, hyperemesis gravidarum.

The first pregnancy was the most severe. At 11 weeks and 2 days, she was hospitalized for hyperemesis gravidarum and inability to eat or drink. After transient improvement with infusion therapy, abdominal pain began approximately 1 h after food intake and became unremitting. Contrast-enhanced abdominal computed tomography at 19 weeks did not identify mesenteric arterial thrombosis or another explanatory abnormality. Owing to unbearable pain and progressive deterioration, with body weight decreasing from 54 to 39 kg, pregnancy was terminated at 19 weeks and 3 days. Mild pain persisted for approximately 2 weeks and then resolved. Similar abdominal colic recurred in 2017, 2019, and 2021, again leading to termination. No definite toxic exposure was identified in her household or workplace; therefore, the occupational background is treated as contextual information rather than a confirmed exposure source.

### Diagnostic assessment

During and between pregnancies, the patient underwent extensive investigations, including abdominal ultrasonography, gastroscopy, colonoscopy, barium enema, contrast-enhanced abdominal CT, upper gastrointestinal radiography, CT with small-bowel reconstruction, chest imaging, gynecologic assessment, reproductive evaluation, couple karyotyping, autoimmune testing, and antiphospholipid antibody testing. Detailed historical test results are provided in Supplementary Data Sheet 1. Specialty evaluations did not yield a documented alternative diagnosis. These assessments did not identify bowel obstruction, mesenteric large-vessel disease, inflammatory bowel disease, autoimmune enteritis, a chromosomal abnormality, or an obstetric disorder sufficient to explain the recurrent syndrome. A structured differential diagnosis based on the clinical course and investigations is shown in Table 2.

**TABLE 2:** Differential diagnoses, investigations, and interpretation.

Differential diagnosis or mechanism	Reason considered	Evaluation performed	Interpretation in this case
Obstetric emergency or structural obstetric disorder	Severe abdominal pain during pregnancy requires exclusion of obstetric causes.	Repeated obstetric evaluations and ultrasound follow-up; in G3 fetal heart activity was reassuring and abdomen was soft by clinical history.	No obstetric diagnosis sufficient to explain the recurrent 11–12-week syndrome was documented.
Bowel obstruction, inflammatory bowel disease, autoimmune enteritis, or major structural gastrointestinal disease	Colic, constipation, inability to maintain oral intake, and weight loss.	Gastroscopy showed erosive gastritis/carditis; barium enema was normal; colonoscopy showed no colonic abnormality; inflammatory bowel disease antibody panel was unremarkable; CT and small-bowel reconstruction did not identify an explanatory lesion.	Available tests reduced the likelihood of these diagnoses, although intermittent functional or motility disorders cannot be excluded by these tests alone.
Mesenteric vascular disease, ischemic-type bowel-wall episodes, or antiphospholipid-spectrum microvascular disease	Pregnancy is prothrombotic; the patient had a slightly elevated lupus anticoagulant ratio; symptoms were severe and recurrent.	Contrast-enhanced CT did not identify mesenteric arterial thrombosis; APS 12-antibody panel in 2024 was negative; right uterine artery diastolic flow abnormality and thromboelastographic hypercoagulability were noted early in G6; aspirin and fondaparinux were used.	Large-vessel thrombosis was not demonstrated. Microvascular or transient ischemic-type mechanisms were not formally excluded and are an important alternative explanation for improvement in G6.
Acute hepatic porphyria, including acute intermittent porphyria	Hormone-sensitive disorders can cause recurrent severe abdominal pain and constipation and may be quiescent outside pregnancy.	No urine porphobilinogen, delta-aminolevulinic acid, or porphyrin testing during an attack was documented.	Not formally excluded; the phenotype overlaps with acute hepatic porphyria.
Hereditary angioedema, including estrogen-sensitive disease with normal C1-inhibitor	HAE can present with recurrent severe abdominal attacks and can worsen during pregnancy or estrogen exposure.	No formal C4, C1-inhibitor antigen/function, or HAE genetic evaluation was documented.	Not formally excluded. Absence of documented cutaneous swelling, airway edema, or family history lowers suspicion but does not eliminate the diagnosis.
Functional gastrointestinal disorder, intestinal dysmotility, or visceral hypersensitivity	Pregnancy-related hormonal and autonomic changes can exacerbate motility and visceral sensitivity phenotypes.	No formal motility assessment, manometry, or visceral sensitivity testing outside pregnancy was documented.	Not formally excluded; discussed as a plausible non-EDC alternative or cofactor.
Abdominal migraine or centrally mediated episodic abdominal pain syndrome	Recurrent severe abdominal pain without a fixed structural explanation; pregnancy-related neurohormonal changes could alter pain thresholds	No formal neurology assessment, Rome IV-directed evaluation, or specific migraine-oriented workup was documented	Lower probability but unexcluded alternative diagnosis
EDC-related host susceptibility	Markedly elevated creatinine-adjusted MEP, methylparaben, and propylparaben were found after conventional evaluations were unrevealing; these biomarkers declined below laboratory limits after intervention.	First-morning urine biomonitoring by UPLC–MS/MS in 2024 and early-pregnancy repeat testing in 2025.	Clinically actionable, hypothesis-generating clue only. It does not establish that EDCs caused the prior syndrome.

At presentation in 2024, body mass index was 23.31 kg/m^2^. APS 12-antibody testing was negative for standard and non-standard antiphospholipid antibodies; however, a lupus anticoagulant ratio of 1.21 was recorded as slightly elevated. Antinuclear antibody testing and a 15-item autoimmune panel were negative. Additional findings included vitamin D deficiency, elevated homocysteine, insulin resistance, elevated natural killer cell cytotoxicity, and moderate gut dysbiosis.

Exposure-history review included occupational, dietary, household, cosmetic, and environmental sources commonly associated with non-persistent EDCs. The patient worked as a diagnostic sonographer and did not report a definite occupational chemical exposure. Household and dietary review focused on food-contact plastics, heating food in plastic containers, packaged or plastic-contact foods, and other avoidable plastic exposure. Cosmetic and personal-care review focused on fragranced products and non-essential personal-care chemicals, which are recognized potential sources of phthalates and parabens. No single dominant source of exposure was identified; therefore, the elevated biomarkers were interpreted as a nonspecific exposure or susceptibility signal rather than evidence of a defined exposure event.

On 14 September 2024, first-morning urine EDC biomonitoring was performed at Xiong’an Miaoxin Medical Laboratory Co., Ltd., using ultra-performance liquid chromatography–tandem mass spectrometry (UPLC–MS/MS). Results were creatinine-adjusted and reported as μg/g creatinine. Monoethyl phthalate (MEP) was 2136.48 μg/g Cr (laboratory upper reference limit, 197.59 μg/g Cr), methylparaben was 233.40 μg/g Cr (upper reference limit, 37.83 μg/g Cr), and propylparaben was 53.80 μg/g Cr (upper reference limit, 13.02 μg/g Cr). Other measured biomarkers, including monomethyl phthalate, monobutyl phthalate, benzylbutyl phthalate, mono(2-ethylhexyl) phthalate, bisphenol A, ethylparaben, and butylparaben, were below the laboratory upper reference limits or not detected. The laboratory report provided analyte values, units, the assay platform, and laboratory upper reference limits; the reference intervals were laboratory-defined from urine samples of a reference population using percentile-based statistics and clinical interpretation. Full assay-validation metrics and internal quality-control procedures were not available in the clinical report and are therefore not overinterpreted here. The full biomarker panel and assay details are presented in Supplementary Data Sheet 2. These findings shifted the working framework toward excessive maternal exposure, altered metabolism or clearance, or host susceptibility to EDCs as a potentially modifiable clue, not a confirmed etiology.

### Therapeutic intervention

A multidomain preconception intervention was implemented (Table 3 and Supplementary Data Sheet 3). Exposure-reduction counseling emphasized avoidance of heating food in plastic containers, reduced unnecessary contact with plastics, reduction of fragrance and non-essential personal care chemicals, increased hydration, and regular aerobic exercise. Medical and nutritional optimization addressed vitamin D deficiency, hyperhomocysteinemia, insulin resistance, immune/vascular risk, and gut dysbiosis. Because the intervention was multifactorial, the subsequent pregnancy outcome cannot be attributed to any single component, including EDC reduction alone.

**TABLE 3:** Multidomain intervention and causal-interpretation limitations.

Domain	Intervention recorded	Available dose or detail	Comment for causal interpretation
Exposure reduction	Reduced heating food in plastic containers; reduced unnecessary plastic contact; reduced fragranced/non-essential personal-care chemicals; increased hydration and aerobic exercise.	Behavioral counseling; adherence recorded qualitatively.	Could reduce EDC exposure but cannot be separated from other interventions.
Nutritional/metabolic	Vitamin D, folate, methylcobalamin, vitamin B6, metformin.	Vitamin D 2000 IU/day; folate recorded as routine preconception supplementation; metformin 500 mg twice daily; methylcobalamin and vitamin B6 at routine doses.	May have improved vitamin D deficiency, hyperhomocysteinemia, and insulin resistance.
Immune/vascular	Low-dose aspirin and hydroxychloroquine.	Aspirin 50 mg/day; hydroxychloroquine 0.1 g twice daily.	Could independently affect immune or vascular pregnancy biology.
Ovarian/endocrine and mitochondrial support	DHEA and coenzyme Q10.	DHEA 25 mg/day; coenzyme Q10 200 mg/day.	Could influence reproductive or metabolic physiology; role in the pain phenotype is unknown.
Microbiome/gastrointestinal support	Probiotics and compound digestive enzyme tablets.	Probiotics two sachets/day; digestive enzyme tablets per clinical prescription.	Could affect gut symptoms or inflammatory tone; effect cannot be isolated.
Index pregnancy anticoagulation	Fondaparinux after early pregnancy vascular/high-coagulability findings.	Specific dose was not documented.	Major confounder because it may have mitigated a vascular or APS-spectrum mechanism.

### Follow-up and outcomes

**FIGURE 1:**
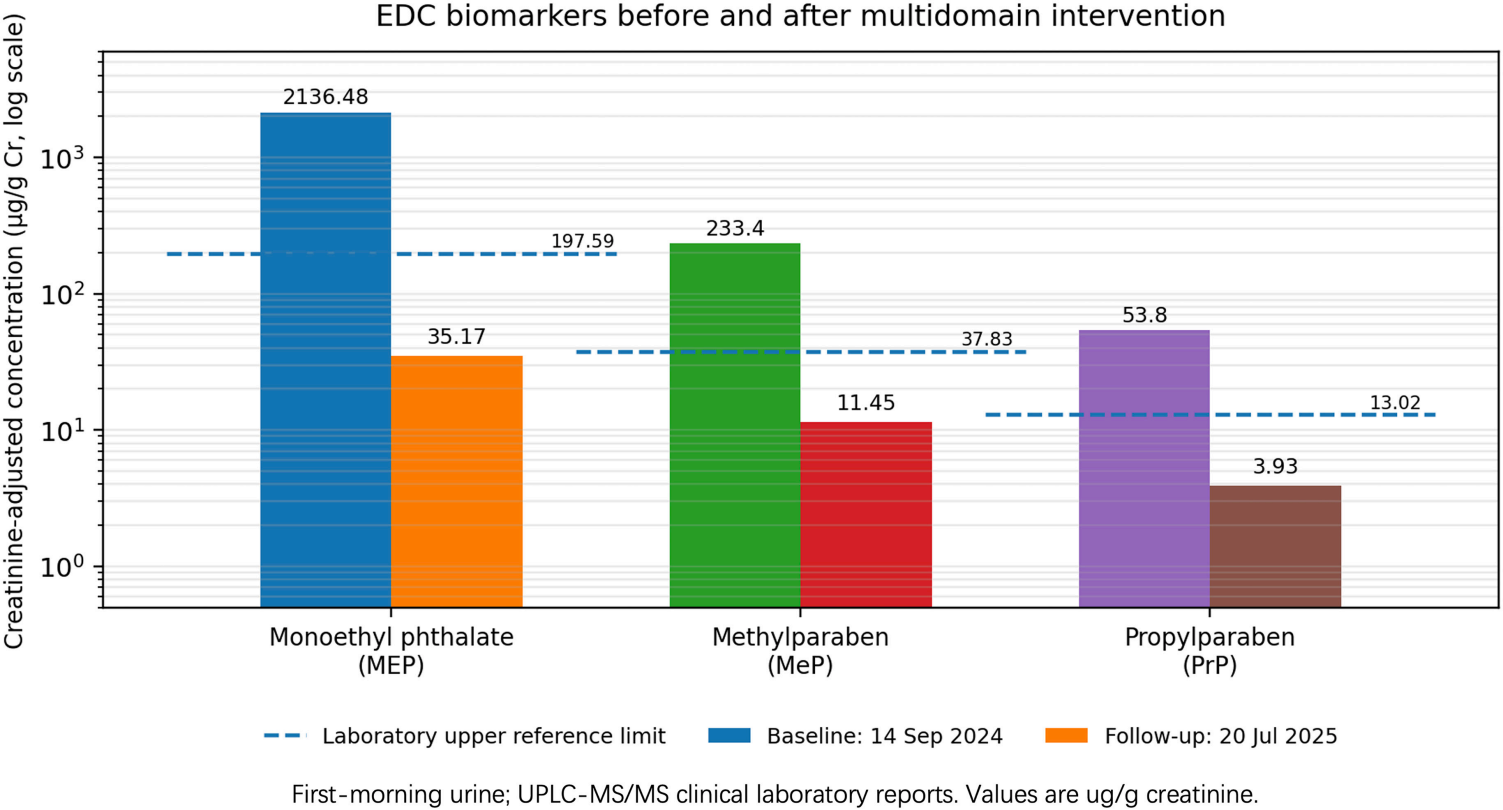
Creatinine-adjusted urinary endocrine-disrupting chemical biomarkers at the two available time points. Bars show first-morning urine concentrations of monoethyl phthalate, methylparaben, and propylparaben measured on 14 September 2024 and 20 July 2025, reported as μg/g creatinine. Dashed horizontal segments denote laboratory upper reference limits. The *y*-axis is logarithmic to allow simultaneous visualization of higher and lower values. Measurements were performed by ultra-performance liquid chromatography–tandem mass spectrometry (UPLC–MS/MS). Figure created by the authors from de-identified clinical laboratory reports.

The patient conceived naturally, with a last menstrual period on 12 June 2025. On 20 July 2025, at approximately 5 gestational weeks and before the historical 11–12-week symptom window, repeat first-morning urine testing showed that the previously elevated biomarkers had decreased below the laboratory upper reference limits: MEP decreased to 35.17 μg/g Cr, methylparaben to 11.45 μg/g Cr, and propylparaben to 3.93 μg/g Cr (Figure 1). Homocysteine and several immune markers also improved. At 6 weeks, ultrasound suggested partial absence of right uterine artery diastolic flow and thromboelastography suggested hypercoagulability; fondaparinux was initiated. At 9 weeks, nausea and vomiting began, but the characteristic abdominal colic did not recur.

She required two hospitalizations for severe hyperemesis gravidarum between 10 and 15 gestational weeks. Clinical features included inability to maintain oral intake, urine ketones up to 4+, weight loss to 49 kg, hypoproteinemia, electrolyte disturbance, and metabolic acidosis. Serum beta-human chorionic gonadotropin (hCG) was 235,175 IU/L at 11 weeks and 5 days and >284,000 IU/L at 12 weeks and 2 days. Thyroid function showed suppressed thyroid-stimulating hormone and elevated FT3/FT4 with negative thyroid antibodies, supporting transient gestational thyrotoxicosis. Treatment included thiamine, intravenous fluids, omeprazole, potassium supplementation, ondansetron, metoclopramide, and ultrasound-guided right subclavian central venous catheterization. The hyperemesis course was consistent with recognized severe nausea and vomiting of pregnancy, and high hCG is a known contributor to transient gestational thyrotoxicosis, although contemporary work also supports placental GDF15 and maternal sensitivity to GDF15 as major determinants of hyperemesis risk (11–13).

**FIGURE 2:**
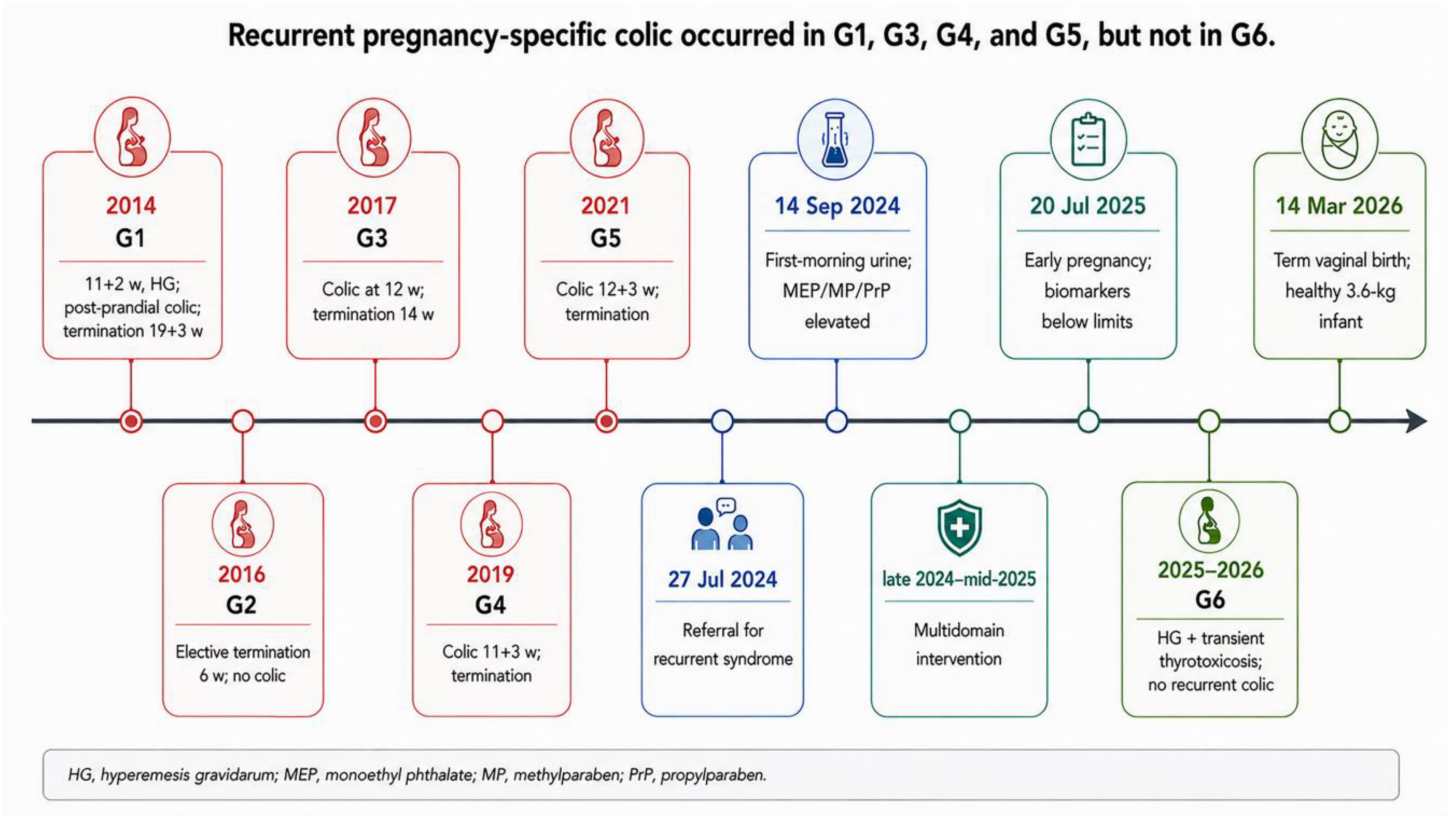
Timeline of obstetric events, symptom recurrence window, investigations, biomonitoring, interventions, and outcomes. A horizontal 16:9 timeline summarizes the six pregnancies (G1–G6), gestational age at onset of the recurrent abdominal-colic phenotype, major diagnostic milestones, urinary EDC biomonitoring dates, multidomain preconception intervention, early-pregnancy vascular findings, and final term live birth. Recurrent severe colic occurred in G1, G3, G4, and G5, typically at 11–12 gestational weeks but did not recur in G6. Figure created by the authors from de-identified clinical data and laboratory reports.

The key within-patient contrast was that the disabling abdominal colic phenotype that had repeatedly appeared at 11–12 weeks in prior pregnancies did not recur. Symptoms of hyperemesis improved after 15 weeks, body weight gradually increased, and prenatal follow-up remained reassuring (Figure 2). On 14 March 2026, she delivered a healthy female infant vaginally at term, weighing 3.6 kg. Maternal weight before delivery was 61 kg. Placental pathology was not performed.

### Patient perspective

The patient provided the following perspective: “In my previous pregnancies, unbearable abdominal pain repeatedly appeared at almost the same gestational period and eventually forced termination. This was physically and emotionally devastating. After a comprehensive preconception evaluation and intervention, I still experienced severe hyperemesis in the successful pregnancy, but the previous abdominal colic did not return, and I finally delivered a healthy baby. I hope sharing this experience will provide clues for other patients with similarly unexplained pregnancy-limited symptoms.”

## Discussion

This case is clinically informative because it describes a recurrent, gestation-associated abdominal pain phenotype that was severe, reproducible, and difficult to classify within conventional obstetric or gastrointestinal diagnoses. Across four prior symptomatic pregnancies, the patient developed disabling lower or left lower abdominal colic, constipation, inability to maintain oral intake, and marked weight loss at a similar gestational window of approximately 11–12 weeks; symptoms were absent outside pregnancy and resolved within 1–2 weeks after pregnancy termination. Repeated conventional assessments did not identify a structural gastrointestinal, large-vessel vascular, autoimmune, chromosomal, or obstetric diagnosis sufficient to explain the recurrent syndrome. In the subsequent pregnancy, after multidomain preconception intervention and marked decline of three creatinine-adjusted urinary EDC biomarkers, the patient still developed severe hyperemesis gravidarum and transient gestational thyrotoxicosis, but the previous abdominal colic phenotype did not recur and the pregnancy ended in term live birth. This within-patient contrast is clinically striking, but it should not be interpreted as proof that EDC reduction caused the changed pregnancy course.

The distinction between the historical abdominal colic phenotype and hyperemesis gravidarum in the successful pregnancy is important. During the index pregnancy, the patient had severe nausea and vomiting, ketonuria, electrolyte disturbance, high hCG concentrations, and transient gestational thyrotoxicosis. These findings are consistent with recognized physiology linking hCG with gestational thyroid stimulation and vomiting severity, while contemporary evidence also implicates placental GDF15 biology and maternal sensitivity to GDF15 in susceptibility to nausea and vomiting of pregnancy (11–13). By contrast, the previous recurrent syndrome was dominated by post-prandial intractable colic, constipation, inability to eat, and catastrophic weight loss, repeatedly leading to pregnancy termination. The absence of that pain phenotype in the index pregnancy suggests that the earlier syndrome and the later hyperemesis course may have shared pregnancy-related triggers but were not clinically identical.

Endocrine-disrupting chemicals remain biologically plausible only at the level of hypothesis. Phthalates and parabens can interfere with endocrine signaling, reproductive hormone dynamics, immune regulation, oxidative balance, and placental biology (2, 3). Personal-care product use during pregnancy has been associated with urinary phthalate and paraben concentrations, and product substitution can reduce several urinary biomarkers, including methylparaben and propylparaben (4, 5). Systematic review and cohort data suggest associations between phthalate exposure and several female reproductive and developmental outcomes, while also emphasizing heterogeneity and limitations in exposure assessment (6–8). Evidence for parabens and female reproductive health remains more limited and sometimes inconsistent, although epidemiologic and experimental studies raise concerns regarding fertility, gestational length, birth weight, hormone levels, and reproductive tissue signaling (9, 10). However, direct human mechanistic evidence linking phthalate or paraben biomarkers specifically to severe gestation-limited abdominal colic, gastrointestinal dysmotility, or visceral hypersensitivity is limited. Therefore, any mechanistic bridge between the elevated urinary biomarkers and the prior pain phenotype in this patient must remain speculative.

This case differs from most published reproductive EDC-related outcomes because the clinical endpoint was not infertility, spontaneous miscarriage, fetal anomaly, or preterm birth, but recurrent maternal intolerance of pregnancy due to severe unexplained abdominal colic. Several non-exclusive mechanisms could be considered, including pregnancy-related placental endocrine transition, smooth-muscle dysmotility, visceral hypersensitivity, inflammatory or oxidative signaling, microbiome disturbance, vascular susceptibility, and host immune-metabolic vulnerability. The patient’s insulin resistance, hyperhomocysteinemia, vitamin D deficiency, elevated natural killer cell cytotoxicity, and gut dysbiosis may have lowered the threshold for symptom expression. These possibilities should be regarded as hypotheses rather than established mechanisms. The structured differential-diagnosis framework in Table 2 is therefore essential for maintaining a conservative interpretation.

The most important limitation is irreducible confounding. The intervention included exposure-reduction counseling as well as vitamin D, folate, methylcobalamin, vitamin B6, aspirin, metformin, hydroxychloroquine, DHEA, coenzyme Q10, probiotics, digestive enzyme support, and later fondaparinux during the index pregnancy (Table 3). EDC reduction alone cannot be isolated from these concurrent measures. Metformin may have improved insulin resistance and the metabolic environment. Hydroxychloroquine may have contributed immunomodulatory effects. DHEA may have affected adrenal or reproductive hormone dynamics. Vitamin D, folate, methylcobalamin, and vitamin B6 may have improved nutritional and one-carbon metabolism factors. Probiotics and digestive enzyme support may have influenced gut microbial or gastrointestinal factors. Low-dose aspirin and fondaparinux represent clinically meaningful vascular and anticoagulant interventions. Therefore, any of these interventions, their combination, spontaneous interpregnancy variability, regression to the mean, altered maternal physiology over time, or an unidentified confounder could have contributed to the changed pregnancy course.

The slightly elevated lupus anticoagulant ratio and vascular findings in the index pregnancy require particular caution. Although the APS 12-antibody panel was negative and contrast-enhanced abdominal CT did not identify mesenteric arterial thrombosis, microvascular or transient ischemic-type bowel-wall events were not formally excluded. The early index pregnancy also included right uterine artery diastolic-flow abnormality and thromboelastographic hypercoagulability, followed by fondaparinux treatment. The use of low-dose aspirin before conception and fondaparinux after early pregnancy hypercoagulability findings could have modified a vascular or antiphospholipid-spectrum mechanism, even if the patient did not meet formal APS classification criteria. This alternative explanation has stronger clinical precedent in recurrent pregnancy morbidity than the EDC hypothesis and must remain central to interpretation (14, 15).

The pregnancy-specific nature of the syndrome is also not fully mechanistically established. The patient had no comparable symptoms outside pregnancy, but formal gastrointestinal motility assessment, manometry, visceral sensitivity testing, Rome IV-directed evaluation, and migraine-oriented assessment were not performed. Functional gastrointestinal disorders, intestinal dysmotility, visceral hypersensitivity, or abdominal migraine-spectrum pain may be exacerbated by pregnancy-related hormonal and autonomic changes and may appear pregnancy-specific clinically. Acute hepatic porphyria, including acute intermittent porphyria, remains an important alternative because it can cause severe abdominal pain and constipation, can be triggered by hormonal changes, and may be quiescent between attacks; urine porphobilinogen, delta-aminolevulinic acid, and porphyrin testing during an attack were not available (16, 17). Hereditary angioedema, including estrogen-sensitive disease with normal C1-inhibitor, can present with recurrent abdominal attacks and may worsen during pregnancy; C4, C1-inhibitor antigen/function, and genetic testing were not available (18, 19). These diagnoses remain unexcluded limitations rather than rejected alternatives.

The biomonitoring results should also be interpreted cautiously. The patient had two available first-morning urine EDC measurements: one after the historical symptomatic pregnancies and one in early pregnancy before the previous 11–12-week symptom window. Although the concentrations were creatinine-adjusted, each time point was based on a single urine sample. Urinary biomarkers of non-persistent chemicals, including phthalates and parabens, can vary substantially over time because of short biological half-lives, episodic exposure, hydration status, and behavioral variation (20–22). Thus, the observed biomarker decline is objective and clinically relevant, but it cannot fully characterize habitual long-term exposure. More importantly, biomarkers were not measured during the historical pain episodes; therefore, it cannot be concluded that elevated EDC exposure preceded or contributed to the onset of the prior pregnancy-specific syndrome.

The exposure assessment has similar limitations. The clinical review considered common occupational, dietary, household, cosmetic, and environmental sources of non-persistent EDCs, including food-contact plastics, heating food in plastic containers, packaged or plastic-contact foods, fragranced products, cosmetics, and non-essential personal-care chemicals. No single dominant source and no definite occupational source were identified. Consequently, the elevated biomarkers should be interpreted as a nonspecific exposure or susceptibility signal rather than evidence of a defined exposure event. The laboratory report provided analyte values, creatinine-adjusted units, assay platform, sampling dates, and laboratory-defined upper reference limits; however, full assay-validation metrics and internal quality-control procedures were not available in the clinical report and are not overinterpreted here.

The strengths of this report include the decade-long within-patient pattern, the reproducible gestational timing of symptoms, repeated negative conventional evaluations, quantified creatinine-adjusted urinary biomarker data at two time points, and documented term delivery after absence of the previous colic phenotype. The limitations are substantial. This is a single case and cannot prove causality. Historical records from the unsuccessful pregnancies did not contain a complete set of serial inflammatory markers, nutritional markers, gastrointestinal motility data, or standardized hospitalization metrics for direct cross-pregnancy comparison. Placental pathology was not performed. Placental histopathology could have provided information on maternal vascular malperfusion, intervillous thrombosis, inflammatory lesions, abnormal placentation, or other placental processes that might have linked vascular, immune, endocrine, or inflammatory mechanisms to the clinical phenotype. Its absence limits mechanistic inference. In addition, no mechanistic biomarkers of motility, visceral sensitivity, oxidative stress, porphyria, bradykinin-mediated angioedema, or placental signaling were collected during the prior symptomatic episodes.

The practical lesson is not that EDCs caused the syndrome, but that targeted exposure history and selective biomonitoring may provide a clinically useful clue in highly selected patients with recurrent, unexplained, pregnancy-specific morbidity after standard evaluation has been exhausted. Counseling should remain balanced. Reducing avoidable exposure to heated plastics, unnecessary fragrances, and heavily packaged or plastic-contact foods is reasonable as part of preconception counseling, but complex obstetric outcomes should not be attributed to EDCs with unwarranted certainty. In patients with similar presentations, broad differential diagnosis remains essential, particularly for vascular/APS-spectrum disease, pregnancy-exacerbated gastrointestinal dysmotility, acute hepatic porphyria, hereditary angioedema, and centrally mediated episodic abdominal pain syndromes.

## Conclusion

A decade-long pattern of recurrent pregnancy-specific intractable abdominal colic and weight loss led to four pregnancy terminations after conventional evaluations were unrevealing. Markedly elevated creatinine-adjusted monoethyl phthalate, methylparaben, and propylparaben were subsequently identified and declined below laboratory upper reference limits after a multidomain preconception program. The next pregnancy was complicated by severe hyperemesis gravidarum and transient gestational thyrotoxicosis but not by recurrent colic and ended in term live birth. This case does not prove that EDCs caused the prior syndrome and cannot isolate EDC reduction from other concurrent interventions or spontaneous variability. Rather, it highlights EDC biomonitoring as a potentially modifiable, hypothesis-generating clue in selected patients with otherwise unexplained recurrent gestational morbidity.

## Data Availability

The original contributions presented in this study are included in this article/Supplementary material, further inquiries can be directed to the corresponding author.
