# Supplementary material for: Case Report: Recurrent gestation-limited intractable abdominal colic with elevated phthalate and paraben biomarkers: a hypothesis-generating observation
Source: Front Med (Lausanne). 2026 Jul 13;13:1875138. doi: 10.3389/fmed.2026.1875138 (PMC13402362; doi:10.3389/fmed.2026.1875138)
Supplement: Supplementary file 1 [file Data_Sheet_1.docx]

**Supplementary Data Sheets**

Manuscript: Case Report: Recurrent gestation-limited intractable abdominal colic with elevated phthalate and paraben biomarkers: a hypothesis-generating observation

# Supplementary Data Sheet 1. Historical diagnostic workup

| **Date / period** | **Pregnancy status** | **Investigation** | **Result** | **Interpretation** |
| --- | --- | --- | --- | --- |
| 2014-2015 | G1 and post-termination | Whole-abdominal contrast-enhanced CT; post-pregnancy gastrointestinal evaluation | No mesenteric arterial thrombosis or explanatory abdominal lesion documented. CEA and immune-related tests at outpatient gastroenterology visit reportedly unremarkable. | No large-vessel vascular or structural explanation identified. |
| 9 Mar 2015 | During/after G1 | Gastroscopy | Erosive gastritis and carditis. | Not sufficient to explain the recurrent, gestation-limited colic phenotype. |
| 29 Apr 2015 | Non-pregnant | Barium enema | No abnormality. | Reduced likelihood of a fixed colonic structural lesion. |
| 2017, G3 | Pregnant | Abdominal CT and upper gastrointestinal radiography | CT not explanatory; hiatal hernia excluded by upper gastrointestinal radiography. | No explanatory lesion documented. |
| 16 Dec 2019 | Non-pregnant | Pelvic contrast-enhanced CT with small-bowel reconstruction | Mild cholecystitis and right renal microlithiasis. | Incidental/non-specific findings; not sufficient to explain recurrent pregnancy-specific colic. |
| 24 Dec 2019 | Non-pregnant | Inflammatory bowel disease antibody panel (8 items) | No abnormality. | Reduced likelihood of inflammatory bowel disease in available serologic evaluation. |
| 19 Aug 2020 | Non-pregnant | Painless colonoscopy | No abnormal finding throughout the colon. | Reduced likelihood of colonic structural/inflammatory disease. |
| May 2023 | Preconception | Couple karyotypes | Normal karyotypes. | No chromosomal explanation for recurrent pregnancy morbidity identified. |
| 28 Jul 2024 | Preconception evaluation | APS 12-antibody panel | Standard and non-standard antiphospholipid antibodies negative. | Does not meet serologic evidence for definite APS; does not fully exclude APS-spectrum or microvascular mechanisms. |
| 2024 evaluation | Preconception evaluation | Lupus anticoagulant ratio | Slightly elevated at 1.21. | Important vascular/APS-spectrum clue; addressed as an alternative explanation. |
| 23 Oct 2025 | Index pregnancy | Abdominal ultrasound | Multiple gallbladder polyps. | Not considered sufficient to explain prior recurrent colic phenotype. |
| Multiple time points | Pregnant and non-pregnant | Specialty evaluations | Evaluations at tertiary hospitals did not yield a formal written alternative diagnosis. | No formal written consultation report was available. |

# Supplementary Data Sheet 2. EDC biomonitoring assay details and results

| **Biomarker** | **Abbrev.** | **Baseline 14 Sep 2024 (μg/g Cr)** | **Upper reference limit (μg/g Cr)** | **Follow-up 20 Jul 2025 (μg/g Cr)** | **Interpretation** |
| --- | --- | --- | --- | --- | --- |
| Monomethyl phthalate | MMP | 11.31 | 37.32 | 26.14 | Below upper reference limit at both time points. |
| Monoethyl phthalate | MEP | 2136.48 | 197.59 | 35.17 | Markedly elevated at baseline; below upper reference limit at follow-up. |
| Monobutyl phthalate | MBP | 201.47 | 408.49 | 162.59 | Below upper reference limit at both time points. |
| Monobenzyl phthalate | MBzP | 0.22 | 0.39 | N/A | Baseline below upper reference limit; follow-up not detected or not reported. |
| Mono(2-ethylhexyl) phthalate | MEHP | 8.58 | 21.94 | N/A | Baseline below upper reference limit; follow-up not detected or not reported. |
| Bisphenol A | BPA | 4.29 | 7.17 | 5.40 | Below upper reference limit at both time points. |
| Bisphenol B | BPB | N/A | 3.02 | N/A | Not detected or not reported. |
| 4-Nonylphenol | 4-NP | N/A | 1.39 | N/A | Not detected or not reported. |
| 4-Octylphenol | 4-OP | N/A | 0.68 | N/A | Not detected or not reported. |
| Diethylstilbestrol | DES | N/A | Not applicable | N/A | Report states item not detected. |
| 17α-Ethinylestradiol | EE2 | N/A | Not applicable | N/A | Report states item not detected. |
| Methylparaben | MeP | 233.40 | 37.83 | 11.45 | Elevated at baseline; below upper reference limit at follow-up. |
| Ethylparaben | EtP | 6.57 | 26.05 | 7.80 | Below upper reference limit at both time points. |
| Propylparaben | PrP | 53.80 | 13.02 | 3.93 | Elevated at baseline; below upper reference limit at follow-up. |
| Butylparaben | BuP | 0.28 | 0.64 | 0.03 | Below upper reference limit at both time points. |

# Supplementary Data Sheet 3. Available intervention details

| **Domain** | **Intervention recorded** | **Available dose or detail** | **Comment for causal interpretation** |
| --- | --- | --- | --- |
| Exposure reduction | Reduced heating food in plastic containers; reduced unnecessary plastic contact; reduced fragranced/non-essential personal-care chemicals; increased hydration and aerobic exercise. | Behavioral counseling; adherence recorded qualitatively. | Could reduce EDC exposure but cannot be separated from other interventions. |
| Nutritional/metabolic | Vitamin D, folate, methylcobalamin, vitamin B6, metformin. | Vitamin D 2000 IU/day; folate recorded as routine preconception supplementation; metformin 500 mg twice daily; methylcobalamin and vitamin B6 at routine doses. | May have improved vitamin D deficiency, hyperhomocysteinemia, and insulin resistance. |
| Immune/vascular | Low-dose aspirin and hydroxychloroquine. | Aspirin 50 mg/day; hydroxychloroquine 0.1 g twice daily. | Could independently affect immune or vascular pregnancy biology. |
| Ovarian/endocrine and mitochondrial support | DHEA and coenzyme Q10. | DHEA 25 mg/day; coenzyme Q10 200 mg/day. | Could influence reproductive or metabolic physiology; role in the pain phenotype is unknown. |
| Microbiome/gastrointestinal support | Probiotics and compound digestive enzyme tablets. | Probiotics two sachets/day; digestive enzyme tablets per clinical prescription. | Could affect gut symptoms or inflammatory tone; effect cannot be isolated. |
| Index pregnancy anticoagulation | Fondaparinux after early pregnancy vascular/high-coagulability findings. | Specific dose was not documented. | Major confounder because it may have mitigated a vascular or APS-spectrum mechanism. |

Note: Folate, methylcobalamin, and vitamin B6 were recorded as routine supplementation. The exact folate unit requires verification against the clinical prescription because the clinical note contains a likely unit inconsistency.
